# Energy-Guided Temporal Segmentation Network for Multimodal Human Action Recognition

**DOI:** 10.3390/s20174673

**Published:** 2020-08-19

**Authors:** Qiang Liu, Enqing Chen, Lei Gao, Chengwu Liang, Hao Liu

**Affiliations:** 1 School of Information Engineering, Zhengzhou University, Zhengzhou 450000, China; a973052438@163.com (Q.L.); liangchengwu0615@126.com (C.L.); 15515732093@163.com (H.L.); 2 Department of Electrical and Computer Engineering, Ryerson University, Toronto, ON M5B 2K3, Canada; iegaolei@gmail.com

**Keywords:** multimodal action recognition, motion energy, temporal segmentation network, heterogeneous convolutional neural networks

## Abstract

To achieve the satisfactory performance of human action recognition, a central task is to address the sub-action sharing problem, especially in similar action classes. Nevertheless, most existing convolutional neural network (CNN)-based action recognition algorithms uniformly divide video into frames and then randomly select the frames as inputs, ignoring the distinct characteristics among different frames. In recent years, depth videos have been increasingly used for action recognition, but most methods merely focus on the spatial information of the different actions without utilizing temporal information. In order to address these issues, a novel energy-guided temporal segmentation method is proposed here, and a multimodal fusion strategy is employed with the proposed segmentation method to construct an energy-guided temporal segmentation network (EGTSN). Specifically, the EGTSN had two parts: energy-guided video segmentation and a multimodal fusion heterogeneous CNN. The proposed solution was evaluated on a public large-scale NTU RGB+D dataset. Comparisons with state-of-the-art methods demonstrate the effectiveness of the proposed network.

## 1. Introduction

As one of the most popular research topics in the field of computer vision, human action recognition has been widely utilized in human–computer interactions, virtual reality, intelligent monitoring, and video retrieval [1,2,3,4]. Though it has recently generated promising performance, video-based action recognition still faces many open challenges [5,6], such as the complexity of action scenes, intra-class differences, and inter-class similarities.

With the popularization of consumer-level depth sensors, video-based action representations can be divided into Red, Green, Blue (RGB) data-based and RGB depth (RGB-D) data-based categories. As a traditional modality for action representation, RGB has more sufficient action appearance information than depth, but it is susceptible to background. As a comparatively new action representation format, depth data not only provide more 3D structure information than RGB data but are also robust to changes in lighting, texture, etc. In addition, skeletal posture information [7] can be obtained from depth video, which provides directions for video-based action recognition. Zhang et al. [8] used a multi-stream neural network joint attribute learner to learn the semantic information between action attributes. Wang et al. [9] proposed a method that extracts three depth sequence representations from a depth map to capture the spatiotemporal characteristics of action. Sahoo et al. [10] extracted a depth history map by estimating the depth map of action frames, and they combined the 3D projection of the depth map for action recognition. Instead of using the temporal information of actions, only the spatial information provided by the depth data is utilized in these methods. Moreover, the research results of traditional action recognition algorithms based on manual feature extraction [11,12,13] have shown that the fusion of RGB and depth data can reduce interference in the background, resulting in better performance. Therefore, the recognition accuracy of actions is greatly improved due to the complementarity of multimodal data. In particular, the misjudgment of similarity actions is well-improved along with the fusion of multimodal information.

Recently, convolutional neural networks (CNNs) have been increasingly applied to action recognition [14,15,16,17,18,19,20,21,22]. The two-stream network is one of the widely utilized methods for human action recognition. Simonyan et al. [14] proposed a two-stream network consisting of a temporal stream and a spatial stream as follows: Static video frames are used as the input of the spatial stream, while dense optical flow frames are taken as the input of the temporal stream; finally, the results of the two streams are fused. Nevertheless, there exists a problem, in that the long-term information of the video is maybe not effectively used by the two-stream network. Wang et al. [15] proposed a long-time modeling temporal segmentation network (TSN), and the strategy is summarized as follows: First, the video is divided into several segments, then the video frames are randomly selected from each small segment and sent to the two-stream network with the purpose of feature extraction and prediction, and, finally, the prediction results of each segment are fused. Some schemes, based on a two-stream network, adopt the TSN’s video segmentation strategy but ignore the difference among the video frames and the distinct characteristics between the temporal and the spatial streams.

**Figure 1:**
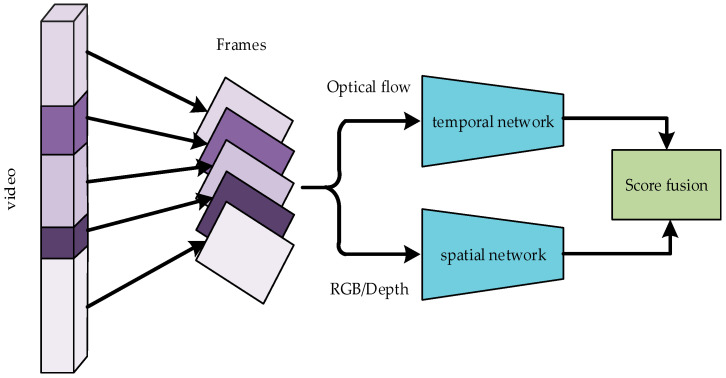
Energy-guided segmented multimodal fusion network (EGTSN): The video is divided into p segments according to the distribution of energy, the frames extracted from each segment are sent to heterogeneous networks, and, finally, all results are merged.

Aiming to address the mentioned above problems, this paper proposes an energy-guided segmented multimodal fusion network (EGTSN). Specifically, the model is composed of energy-guided video sparse time sampling, heterogeneous convolutional neural networks for feature extraction, and a multimodal weighted fusion part. The architecture of EGTSN is shown in Figure 1. The main contributions and novelty of this paper are summarized as follows:

- An effective EGTSN was designed by using the distribution of motion energy extracted from the depth videos to guide segmentation for the purpose of modeling individual action motion stages and solving the problem of underutilized motion information amongst video frames in a TSN.
- By employing spatiotemporal heterogeneous two-stream networks to improve the performance of homogeneous two-stream networks of the TSN, the EGTSN can obtain more sufficient feature representations and reduce the redundancy of the network.
- The traditional optical flow information is used in depth videos to perform multimodal fusion, thus improving the misjudgment of similar actions.

The rest of the paper is organized as follows. A review of related work is presented in Section 2. The EGTSN is proposed in Section 3. Experimental results and analysis are shown in Section 4. Conclusions are drawn in Section 5.

## 2. Related Work

In this section, we briefly review the video-based human action recognition methods, especially in multi-modal based convolutional networks.

Hand-crafted features: Feature extraction algorithms, based on the features of each category of action, are manually designed and use machine learning methods to train and classify the extracted features. Superior performance and stability have been shown in the improved dense trajectory (iDT) [23], which models the motion trajectory in a video through the optical flow field and extracts the features along the trajectory. Hu et al. [24] proposed a joint heterogeneous feature learning method based on RGB and depth data. They used the time Fourier feature to extract the trajectory and spatiotemporal features in the depth; then, the trajectory information in RGB is extracted through the histogram of oriented gradient (HOG) method [25], and, finally, heterogeneous joint training is conducted for a variety of features. A cross-modal feature learning method, in which depth features and RGB visual features are jointly used, was proposed by Kong et al. [11] to classify different actions. Due to various action characteristics, the required hand-crafted features are different. Therefore, hand-crafted feature-based action recognition has less generalization and robustness.

Deep learning: The good performance of a long short-term memory (LSTM) network [26] in dealing with the sequence features problem makes it possible to model the temporal relationship between frames. Donahue et al. [27] combined LSTM with a CNN to produce integrated features. Wu et al. [28] proposed a network that jointly exploits CNNs and LSTM networks. A two-stream CNN is employed to extract action features, and the features are sent into a bidirectional LSTM network to model the time structure. Though the method based on LSTM or the combination of LSTM and the CNN has achieved good results, it still cannot handle the spatiotemporal characteristics of the actions well. Tran et al. [16] proposed a 3D CNN by extending the convolutional and pooling layers from two dimensions to three dimensions, so that convolution is carried out in the temporal and the spatial domains simultaneously. In addition, some variants of 3D CNN, like Discriminative Motion Cues(DMC-Net) [17] and Efficient Convolutional Network (ECO) [18], have achieved better performance, while 3D CNN-based methods cannot model long-range time structures and have expensive computational costs. Tu et al. [19] designed a three-stream CNN based on a two-stream network; here, the spatiotemporal significance of object segmentation is utilized to extract the semantic information related to human actions, then the three-stream CNN is used to process the multimodal derived from the spatiotemporal domain, and, finally, the extracted semantic information related to human actions is integrated into the architecture to improve the accuracy of action recognition. Zolfaghari et al. [20] employed a fast-convolutional network to estimate the human body position, wherein spatiotemporal architecture is applied to obtain the characteristics of action. Though methods based on multi-stream CNNs can effectively obtain the spatiotemporal information of an action, each of them has its limitations.

In this paper, we propose a novel energy-guided temporal segmentation method, named the EGTSN, that can effectively acquire spatiotemporal information and make full use of multimodal complementary characteristics.

## 3. Proposed Method

In order to make better use of key frames with rich action information in videos, it is essential to design segmentation strategies for long-term dynamics inherited with videos. In this section, we first discuss how to extract motion energy and optical flow from depth videos. Then, we explain the architecture of our EGTSN.

### 3.1. Extraction of Motion Energy

Depth images provide more opportunities for traditional action recognition than RGB images, since depth modality has complementary information. In addition, compared with RGB images, depth images have the advantages of insensitivity to lighting, texture, and color changes.

The general method of video segmentation is to evenly divide the video into several parts according to a temporal index. However, our energy-guided segmentation divides video into several segments with the even energy according to the distribution of the whole video motion energy. Therefore, frames with rich motion energy information can provide more motion features for action recognition. Thus, the more motion information a segment contains, the shorter its length will be; as such, the key frames can be easily extracted during random sampling.

A depth video containing K frames is projected into three orthogonal planes in the x, y, and z directions. Three views are generated from these directions—the main, side, and top views, respectively. The motion energy in each view can be represented by the absolute value of the pixel difference between two consecutive frames. Finally, the motion energy of the whole depth video can be obtained by accumulating the motion energy of three views. E(i) denotes the motion energy from frame 1 to frame i, and the whole video energy accumulation process is defined as follows:

(1) $$E\left(i\right)=\sum_{v=1}^{3}\sum_{k=1}^{i-1}\left(sum(\left|F_{v}^{k+1}-F_{v}^{k}\right|)\right)$$

where k is the frame index and v represents the three views. $F_{v}^{k}$ represents the frame k projected on the plane v. $sum(\left|F_{v}^{k+1}-F_{v}^{k}\right|)$ is the sum of the absolute values of pixel differences between two adjacent frames in the same view.

(2) $$E_{N}\left(i\right)=\left\{ \begin{array}{cc} \frac{E\left(i\right)}{E\left(K\right)} & i<K\\ 1 & i=K \end{array}\right.$$

**Figure 2:**
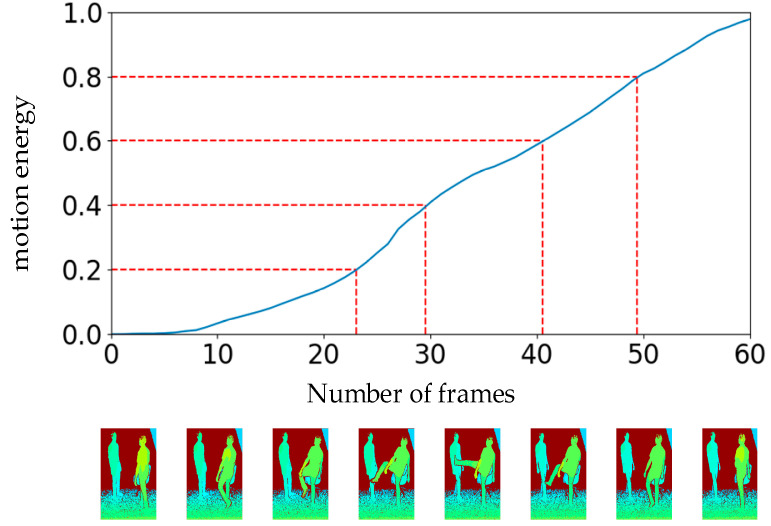
Video uniform segmentation diagram based on the motion energy of depth modality. The abscissa represents the frame number of the video, and the ordinate represents the normalized motion energy. The depth image in this graph is visualized.

The total motion energy of the video is represented by E(K). According to Equation (2), $E_{N}\left(i\right)$ denotes the normalized motion energy. The motion energy accumulation of a kicking action video is shown in Figure 2.

### 3.2. Extraction of Depth Optical Flow

The existing action recognition methods based on depth video generally recognize objects by acquiring object positioning from the depth video and extracting motion history maps or skeleton information as action features. These operations make more use of the spatial information of the actions in the depth data while ignoring the temporal information of the actions. The traditional method of extracting optical flow from RGB data is to first gray the RGB image and then use the optical flow algorithm to extract the optical flow from the grayscale image. Inspired by this idea, this paper proposes an optical flow extraction method based on depth video that is combined with the characteristics of depth data.

The distance between the object and the depth sensor is decided by the pixel value in the depth video frame, thus reflecting the spatial information of the target. The pixel values in most areas of the depth frame are zero due to the limitation of depth sensor sensitivity. From another point of view, background interference to the foreground is reduced by this situation, which also greatly reduces the amount of calculation for our quantization operation. The range of pixel values is from 0 to 8000, after the analysis of the NTU RGB+D [29] dataset. Based on the discussion above, in our method, we uniformly quantized the depth map so that the range of the pixel value was from 0 to 255.

**Figure 3:**
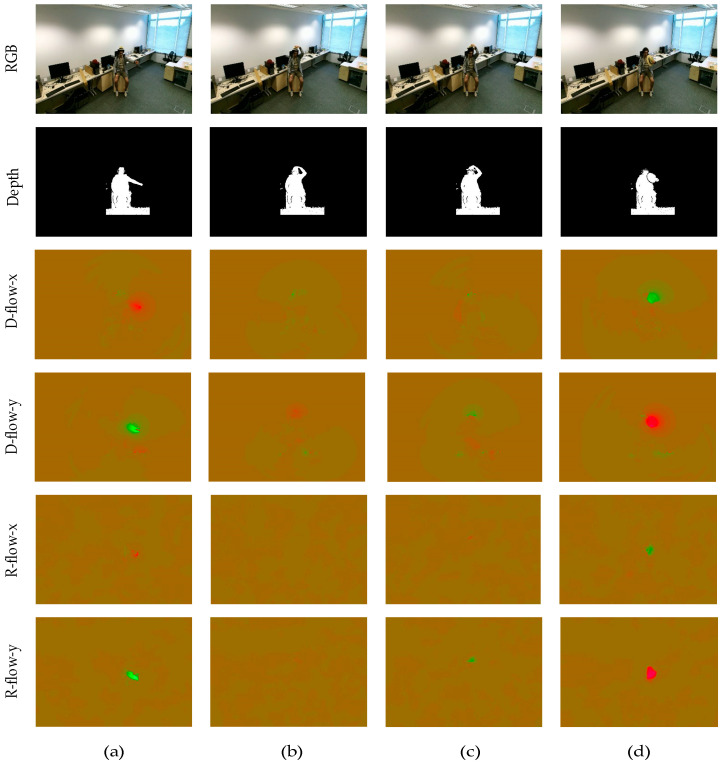
A schematic diagram of four-modal action video frames. (**a**–**d**) represent the 20th, 40th, 60th, and 80th frames of the current action, respectively. For the purpose of clarity, pseudo-color processing was performed on the images of two optical flows.

After quantizing the depth video, the optical flow was calculated by using the total variation (TV) regularization and the robust L1 norm (TV-L1) optical flow extraction algorithm found in [30]. The difference in the pixel value range of different datasets was caused by the difference in the sensitivity of the depth sensor, but it is quantified to the same range before the optical flow is extracted in our method. Therefore, this deep optical flow extraction method is accessible for other datasets. The optical flow extracted from RGB is denoted as R-flow, while the optical flow extracted from depth is denoted as D-flow. Taking the action of removing the hat as an example, Figure 3 lists a partial frame diagram of the four-modal data of RGB, depth binarization, R-flow, and D-flow. For the sake of clarity, the images of two optical flows were processed with pseudo color. It can be seen from Figure 3 that D-flow was similar to R-flow in the x and y directions, and the effectiveness of D-flow in multimodal action recognition was seen in experimental results.

### 3.3. Energy-Guided Temporal Segmentation

Modeling the long-range time structure of an action is a challenging in trimmed action videos. Wang et al. [15] proposed a temporal segmentation network using sparse time sampling and video-level supervision strategies so that the action recognition effect in the entire video range can be effectively improved. However, the action information in each video frame is treated equally. However, how to locate the timing position of the action is particularly important in the untrimmed video. Lin et al. [31] proposed an effective proposal generation method called a boundary-sensitive network (BSN). A BSN generates a good performance by locating the beginning, continuation, and end stages of an action in an untrimmed video to support subsequent action recognition tasks. This paper focuses on sub-action segmentation of actions in trimmed videos.

**Figure 4:**
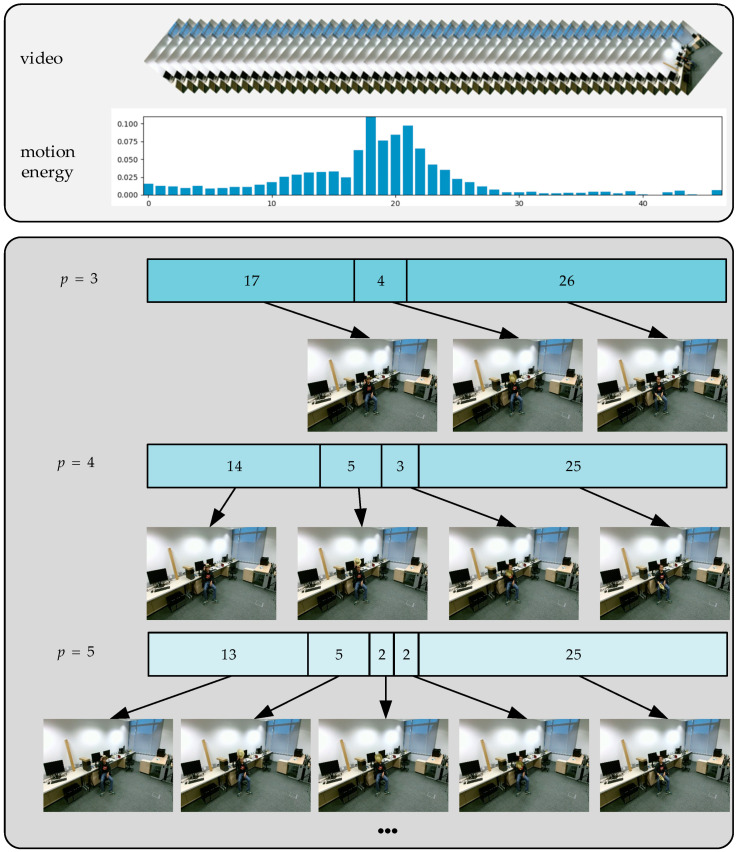
The motion energy distribution of taking off a hat, as well as the temporal segmentation effect of different p values.

For this paper, we focused more on frames with higher motion energy. More detailed information is given as follows: First, suppose a give video V; we extract motion energy from its corresponding depth video and then evenly divide it into p segments $\left\{V_{1},V_{2},\cdots ,V_{p}\right\}$ according to the motion energy distribution. Finally, we randomly sample one frame from each segment to form a short clip $\left\{s_{1},s_{2},\cdots ,s_{p}\right\}$. For example, the motion energy distribution of taking off a hat with 47 frames and the selection of p are shown in Figure 4. It is obvious that the motion energy of taking off the hat was concentrated in the middle of the video, and the larger p was, the more energy-rich frames could be sampled. In other words, the more motion information of each frame in a segment contains, the shorter the segment length will be. Therefore, the key frames can be easily extracted during random sampling. However, with the increase of p, performance tends to be convergent, thus, providing a solution to find the appropriate p on the evaluated dataset.

Afterwards, a video frame $s_{i}$ is sent to a convolutional network to get the action classification score. Then, the average fusion is used to fuse the action category scores of p video frames. Finally, the fusion result is sent to a SoftMax layer to obtain the final classification result. The process of fusion can be modeled as follows:

(3) $$f\left(s_{1},s_{2},\cdots ,s_{p}\right)=S\left(M\left(\sum_{i=1}^{p}F\left(s_{i};W\right)\right)\right)$$

where M is the representation of mean fusion function, $F(s_{i};W)$ represents a convolutional network with parameters W, and S is the SoftMax function.

The loss function of the fusion is defined as follows:

(4) $$L\left(y,m\right)=-\sum_{i=1}^{n}y_{i}\left(m_{i}-\log\sum_{j=1}^{n}\exp m_{j}\right)$$

where $y_{i}$ denotes the ground-truth label of class i, n represents the total number of categories in the dataset, and $m_{i}=M\left(F_{i}\left(s_{1};W\right),F_{i}\left(s_{2};W\right),\cdots ,F_{i}\left(s_{p};W\right)\right)$ indicates the prediction of class i. During the backpropagation process, the gradient of the loss value L relative to the network parameter W is derived as:

(5) $$\frac{\partial L(y,m)}{\partial W}=\frac{\partial L}{\partial m}\sum_{i=1}^{p}\frac{\partial M}{\partial F\left(s_{i}\right)}\frac{\partial F\left(s_{i}\right)}{\partial W}$$

According to Equation (5), the backpropagation uses the results of p clips. Then, the parameters of the network can be updated with a mini-batch stochastic gradient descent.

### 3.4. Multimodal Heterogeneous Networks

Generally, the actions in a video are understood in two ways. One is to extract spatial information from video frames, and the other is to obtain temporal information from the context of video frames. Based on this point, Simonyan et al. [14] proposed a two-stream network that uses temporal and spatial networks to recognize actions. The temporal network is used to extract the action information in the video, and the spatial network is employed to obtain information such as the position, shape, and structure of the object. In a TSN, the network achieves optimal performance when an Inception architecture with Batch Normalization (BN-Inception) is applied to both the spatial and temporal streams at the same time.

**Figure 5:**
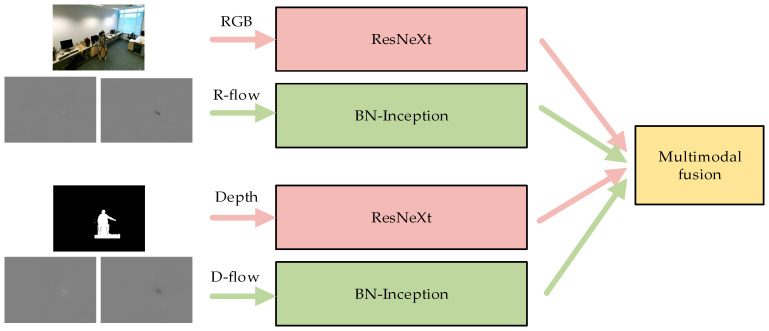
Multimodal heterogeneous networks for the proposed EGTSN model.

However, it is known that the observation and recognition of target shapes and actions are two completely different processes. Therefore, an appropriate network structure for temporal and spatial networks should be designed independently. Previous studies [21,32] have shown that a convolutional neural network with more layers can extract more features and high-level semantic information than a basic network. Nevertheless, as the number of layers increases, the network performance degrades. As a result, the problems of gradient disappearance and gradient explosion may occur, which limits the application of BN-Inception. The outline of the network structure of BN-Inception can be found in Figure 5 of [33]. The proposed Residual Network (ResNet) [21] solves the problems of network degradation and gradient disappearance, and it enables the network to be continuously deepened. The outline of the network structure of ResNet can be found in Figure 3 of [21]. Xie et al. [22] proposed a more excellent ResNeXt network based on ResNet. Even with the same number of convolutional layers, ResNeXt has better performance and fewer parameters than ResNet. The outline of the network structure of ResNeXt can be found in Figure 1 of [22]. According to experimental results in Section 4.3.2, ResNeXt was selected as the convolution architecture of the spatial networks and BN-Inception was chosen as the convolution architecture of the temporal networks. The spatial stream networks deal with RGB and depth data separately, while the temporal stream networks process R-flow and D-flow data separately. In the course of multimodal weighted fusion, the video data of each modality are sent to the trained network to obtain the initial motion classification. Then, a certain weighting coefficient is assigned to the action classification score of each modality before the weighted fusion of the multimodal data classification results is performed. Meanwhile, the optimal weighting factor is obtained by a traversal search. The fusion weights are determined by the data distribution of different datasets. Therefore, the optimal weight obtained is the optimal value on this dataset. Our multimodal heterogeneous networks are shown in Figure 5.

There are two main advantages of using heterogeneous convolutional networks for multimodal action recognition. Firstly, heterogeneous networks can effectively reduce redundant information and improve the utilization accuracy of a model. Secondly, multimodal data are complementary, which can significantly reduce misjudgment for similar actions. This paper verifies the advantages of a heterogeneous model over a homogeneous model in temporal and spatial networks via the experiments in the next section.

## 4. Experiments

In this section, the dataset and experimental settings are first introduced. Afterwards, the performance of the EGTSN is evaluated. Lastly, a comparison with the state-of-the-art is conducted. The hardware platform used in the experiment included an Intel E5-2620 v4 CPU and two NVIDIA Tesla k80 GPUs. Meanwhile, a Python development environment and a PyTorch platform were involved in the software platform.

### 4.1. Evaluation Dataset

**Figure 6:**
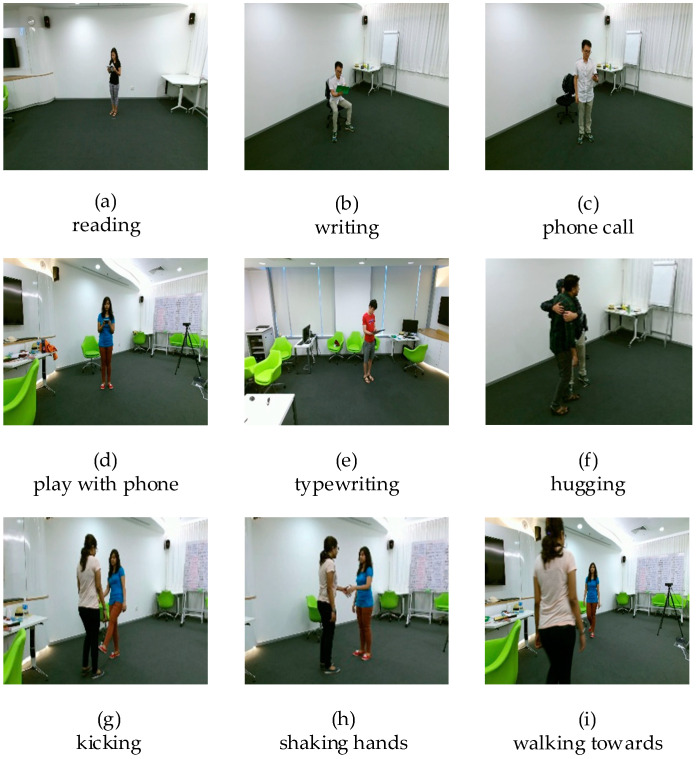
Schematic diagram of some actions of the NTU RGB+D dataset. (**a**–**e**) are similar actions that are difficult to distinguish. (**f**–**i**) represent multi-person interactive actions.

To demonstrate the effectiveness of our method, the NTU RGB+D [29] dataset was used to evaluate the proposed network. The NTU RGB+D dataset consists of RGB, depth, 3D skeleton, and infrared videos provided by the Microsoft Kinect v2 sensor [29,30]. Among the 60 types of actions from 40 different subjects, there are 40 types of daily behaviors (playing with mobile phones, taking off shoes, wearing glasses, etc.), nine kinds of health-related movements (sneezing, vomiting, touching the back, etc.), and 11 kinds of mutual interaction (kicking, hugging, shaking hands, etc.). A total of 56,880 samples were collected. Figure 6 lists some action fragments that contained highly similar actions that were difficult to distinguish in the dataset, such as writing, reading, making a phone call, and playing with a mobile phone. The NTU RGB+D dataset has two protocols to evaluate performance, i.e., cross subject (CS) and cross view (CV) settings. Because the resolution of RGB frames is 1920 × 1080, we resized them to 224 × 224 to match the input size of the convolutional neural networks. In the cross-subject evaluation protocol, the training and testing sets contained data from 20 different subjects. The data of subjects’ ID 1, 2, 4, 5, 8, 9, 13, 14, 15, 16, 17, 18, 19, 25, 27, 28, 31, 34, 35, and 38 were used for training, and the rest were for testing.

### 4.2. Experimental Setup

Pre-training strategy: A large number of studies have shown that the use of pre-trained network parameters on ImageNet [34] can accelerate the convergence and improve the performance of CNNs. We initialized the weights of the heterogeneous networks with pre-trained parameters from ImageNet. Since the size of the first convolution layer Conv1 of the pre-trained model on ImageNet was (64, 3, 7, 7), the RGB stream network weights could be directly initialized using the pre-trained ResNeXt model. However, since the channel number of depth and the two optical flows was not 3, the parameters of the first convolutional layer Conv1 of the pre-training model needed to be preprocessed as follows. As depth data comprised one channel, the new convolution kernel size (64, 1, 7, 7) was obtained by averaging the three channels of Conv1’s convolution kernel. The optical flow data were divided into horizontal and vertical directions, and a continuous five frames of optical flow were used as input to the temporal network; thus, the three channels of Conv1’s convolution kernel were averaged and then copied into 10 copies to obtain a new convolution kernel size (64, 10, 7, 7). Then, these modified weight models were utilized to initialize the heterogeneous networks.

Training: The mini-batch stochastic gradient descent method was used to accelerate the training of the network. The batch size of the spatial network and the temporal network were set to 16 and 32, respectively. The Stochastic Gradient Descent (SGD) optimization algorithm was used to update the parameters of the network. Furthermore, the momentum and weight decay were set as 0.9 and 0.0005, respectively. For the spatial network, we adopted an initial learning rate of 0.001 and a total episode of 100. Then, we decreased the learning rate by 10 times when the episodes were 50 and 80, respectively. For the temporal network, the episode was set as 120. The initial learning rate was set as 0.001, which was decreased by 10 times after 60 and 90 episodes, as well. To prevent overfitting, the dropout of the two streams was set to 0.8 and 0.7. In addition, multi-cropping, horizontal flipping, and scale jittering [31] were used to augment the dataset.

Testing: We combined the original two-stream network test scheme [14] according to the characteristics of energy segmentation. The test video was divided into five segments, on average, according to the motion energy, and for each segment, five frames are sparsely sampled. Then, the sampled video frames were clipped at four corners and one center, again flipped horizontally, and the processed video frames were sent to the trained network. In our approach, average fusion was used to fuse the outputs of a convolutional neural network. The weighted average strategy was adopted to fuse the classification results of multimodal data. In order to achieve optimal performance, the weight value of multimodal fusion was obtained by a traversal search.

### 4.3. Experimental Results and Analysis

In this subsection, we first verify the effectiveness of energy-guided temporal segmentation. Then we conduct a comparative analysis of different network architectures. In addition, we analyze the effect of multimodal fusion on similar actions. Finally, we compare the proposed method with existing methods to verify the effectiveness of the proposed method.

#### 4.3.1. The Effect of Energy-Guided Temporal Segmentation

Before conducting experiments to verify the proposed method, we needed to determine the number of segments for video segmentation. The appropriate number of segments has an important influence on the recognition effect. A segmentation strategy is not used when the value is 1. Additionally, we found that the effect of action recognition kept increasing as segments increased. When the number of segments exceeded five, the recognition effect tended to be saturated. Therefore, the number of video segments was selected as five. All the experiments below are based on this situation.

The TSN divided the video evenly according to the length of the video. This division method has no emphasis, and the action information contained in each frame of the video should be different. In traditional action recognition, motion energy is a good representation of the action information content in video frames. We used motion energy to guide video segmentation so that the network could focus on frames with rich motion energy. To verify the effectiveness of energy-guided temporal segmentation in the model, our method was compared with the TSN method on different data modalities. For the sake of making a fair comparison, the other experimental conditions were set exactly as the same, except for the different ways of video segmentation. The convolutional neural network used in this experiment was ResNeXt101. The experimental results are shown in Table 1. The results show that our method performed better than that of the original TSN method under the same conditions.

#### 4.3.2. The Effect of Heterogeneous Networks

Most existing methods based on spatiotemporal networks use homogeneous CNNs. With the propose of extracting more action features from video frames, generally speaking, deeper CNNs can achieve better effects. However, since the data processed by temporal and spatial networks are different, using the same CNN is not the best choice. In this case, some CNN architectures are good at processing RGB data but unsatisfactory at processing optical stream data. Alternatively, some CNN architectures have greater feature extraction capabilities than RGB for optical flow. Therefore, we believe that heterogeneous networks are more suitable to extract action features from different modal data. We conducted comparative experiments on three convolutional networks, namely BN-Inception, ResNet, and ResNeXt. The experimental results are summarized in Table 2.

It can be seen from Table 2 that the ResNeXt achieved better effects on the spatial network. As a result, we fixed the convolutional network of the spatial stream as ResNeXt. For the temporal stream, as the BN-Inception network obtained the best recognition accuracy on the two optical flow data sets, we fixed the temporal stream convolution network to BN-Inception. According to the experimental results, the performance of the overall spatial network was worse than that of the temporal network. The main reason for this was that the convolutional network was not good at processing small targets, but the area of the action occurrence took up only a small part of the image in the NTU RGB+D dataset, and this area became smaller after performing the operation of changing the size. Additionally, there were a large number of spatially similar videos in the dataset, but the actions of these videos were sequential in the temporal domain, which made it easier for the temporal network to distinguish them well.

Furthermore, depth data can typically be very noisy and have holes due to projection shadows. Fortunately, we did not find any holes in the NTU RGB+D dataset. Thus, we directly extracted the optical flow from depth and RGB. Of course, if there are some holes in depth data, we think the bilinear interpolation should be a good way to mitigate their effects. However, the noise in depth videos is more intense than that in RGB here, so the performance of D-flow was worse than that of R-flow.

#### 4.3.3. The Effect of Multimodal Fusion

To verify the effectiveness of our multimodal fusion, we firstly fused the classification results of RGB and R-flow, and then we moved onto the results of depth and D-flow. Finally, all data modalities were fused. For the selection of the fusion method, the most convenient weighted score fusion was adopted, while the fusion weight was obtained by a traversal search. For the fusion of RGB and R-flow, the fusion weight was set to 1:1, and then the weight of R-flow gradually increased in steps of 0.1. Finally, the optimal fusion weight of RGB and R-flow were frozen to be 1:3. Similarly, we obtained the fusion weights of depth and D-flow as 1:2.9, and the fusion weights of RGB, depth, R-flow, and D-flow were obtained as 1:1.1:3.8:2.1. The final fusion results are summarized in Table 3. As can be seen from the results in Table 3, the fusion of the temporal and spatial streams was much better than the results of the single-modal data. After fusing all the modal data, the accuracy of action recognition was improved and reached its maximum.

**Figure 7:**
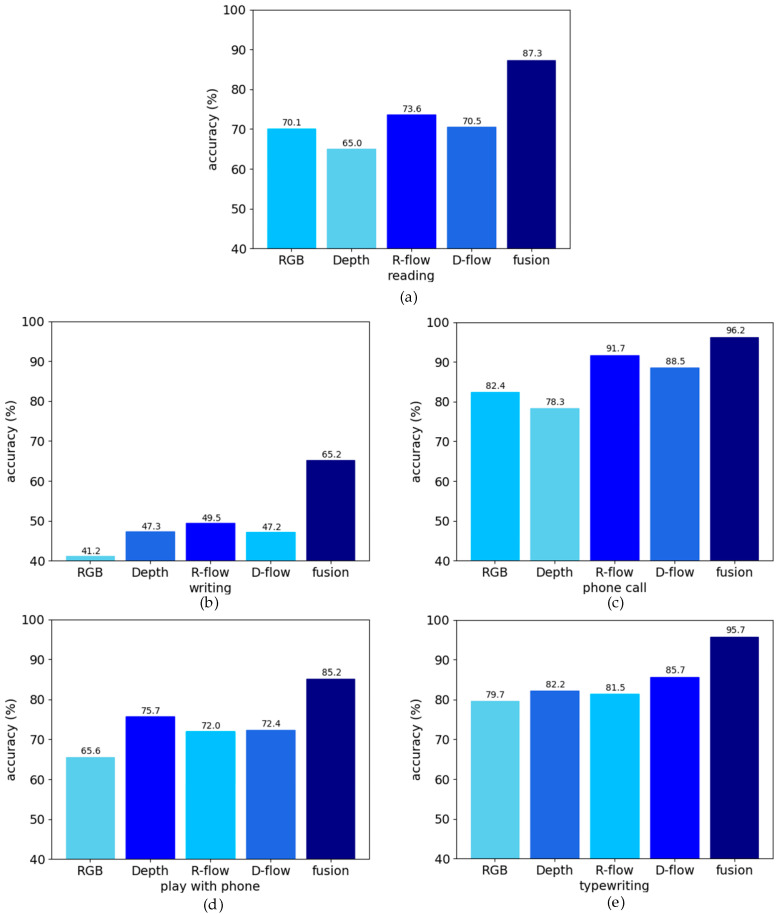
The improvement effect of multimodal fusion on the misjudgment of similar actions.

In addition, from the experimental results, it could be seen that the complementary multimodal data could improve the misjudgment of similar actions. Figure 7 lists the results of the improved recognition performance of multimodal fusion on several similar actions in the NTU RGB+D dataset (such as reading, writing, making phone calls, playing with a phone, and typewriting). This similarity action was embodied by the spatial and temporal domains. In the spatial domain, the relative positions in different actions between the actors and objects were similar to each other, and the appearance of the action was similar to other actions as well. In the temporal domain, the long-lasting states of different actions were similar. For example, writing and playing with a phone were in the same motion state for a long time, so they were prone to being misjudged as each other. The actions of putting on a coat and taking off a coat in the dataset were very similar in the spatial domain, but there was a sequential relation of the actions in the temporal domain. Thus, they could be easily distinguished by heterogeneous networks. In Figure 7, the recognition accuracy from low to high is represented by color from light to deep. Reading and writing were similar actions, and it was easy for them to be misjudged as each other. From Figure 7, it can be seen that multimodal fusion improved the recognition accuracy of these two actions by 10–24%. For several other similar actions, the recognition accuracy also increased by more than 10%. The experimental results validated the performance of our multimodal fusion strategy.

#### 4.3.4. Comparison with Other Methods

This section compares our EGTSN with seven other existing methods, including one method based on single-mode skeleton information and six methods based on multimodal fusion. (1) Wang et al. [35] proposed a method of a joint trajectory map (Jtm) that encodes the spatiotemporal information in the skeleton sequence into multiple images; (2) Zolfaghari et al. [20] proposed a three-stream architecture that integrates posture, motion, and raw images; (3) Wang et al. [9] proposed three representations of depth sequences, namely dynamic depth images (DDI), dynamic depth normal images (DDNI), and dynamic depth motion normal images (DDMNI), which used for isolated and continuous action recognition; (4) Han et al. [36] proposed an artificial prompt two-stream LSTM model based on RGB-D data; (5) Wang et al. [37] proposed an effective fully convolutional network called MF-NET to process multiple input features, and they proposed a novel fusion strategy to combine independent feature learning and dependent feature relating; (6) Li et al. [38] proposed a multi-stream enhanced spatiotemporal graph convolutional network (MS-ESTGCN), where densely connected multiple temporal graph convolution layers (GCLs) with different kernel sizes are applied to aggregate more temporal features; and (7) Zhu et al. [39] proposed an action machine method that separates the human body from the environment and integrates RGB and posture.

The different accuracies of action recognition, obtained by using different schemes, is shown in Table 4. In Table 4, we can see that our approach is superior to most of the state-of-the-art methods, such as the Jtm [35], DDI [9] methods based on convolutional networks, and the Fused View-Invariant and Difference Handcrafted cues (FVDN) [36] method based on two-stream LSTM. Furthermore, compared with the Jtm algorithm, our method’s accuracy was improved by 21.3%. Our accuracy was increased by 6.9% compared with the result of the fusion of the three representations based on the depth map, DDI, DDNI, and DDMNI [10]. Then, compared with the MS-ESTGCN [38] method of fusing six modal data, the accuracy was also improved by 3.3%. Moreover, it was observed that our EGTSN approach achieved a better performance than that of the action machine [39], which also integrates multimodal information to improve its final performance. Note that, in contrast to research focused on detecting the region of action occurrence by an action machine, the focus of EGTSN is on segmenting the videos according to the distribution of motion energy.

In summary, the experimental results showed the advantages of our EGTSN for human action recognition. This was primarily because our EGTSN not only enables the network to pay more attention to key frames but also to better utilize the advantages of multimodal information complementation.

## 5. Conclusions

In this paper, the proposed network of motion energy-guided temporal segmentation, fully mining the spatiotemporal information of actions in multimodal data, was found to significantly improve the performance of human action recognition. Concretely speaking, the motion energy information of a video is used to guide its segmentation and sampling so that the model pays more attention to the frames with rich motion energy. The potential of a CNN’s feature extraction is released by heterogeneous convolutional networks; as such, more spatiotemporal features can be mined. By extracting optical flow information from a depth map, multimodal action recognition reduces the misjudgment of similar actions and improves the accuracy of action recognition. Experiments on the NTU RGB+D dataset verified the performance of our method. In future work, we will try to use deep reinforcement learning to search for key frames that contain rich action information instead of just using motion energy to extract key frames. Additionally, an adaptive weighting method will be employed to assign the weight of multimodal fusion. Thus, the performance of human action recognition will be further improved.

## Figures and Tables

**Table 1:** Recognition accuracy of different video segmentation methods. TSN: temporal segmentation network.

Method	RGB	Depth
TSN	78.4	81.1
Energy-guided	79.6	82.1

**Table 2:** Recognition accuracy of different convolutional network architectures. R-flow: optical flow extracted from RGB; D-flow: optical flow extracted from depth.

CNN	RGB	Depth	R-Flow	D-Flow
Batch Normalization (BN)-Inception	76.9	79.5	90.2	89.6
Residual Network-101 (ResNet101)	78.1	81.4	83.3	84.2
ResNeXt101	79.6	82.1	84.7	85.5

**Table 3:** Recognition accuracy of multimodal fusion.

Data	Accuracy
RGB	79.6
Depth	82.1
R-flow	90.2
D-flow	89.6
RGB and R-flow	92.9
Depth and D-flow	91.7
Multimodal fusion (EGTSN)	94.7

**Table 4:** Comparison between the method proposed in this paper and existing methods.

Method	Accuracy (cs)
Joint Trajectory Maps (Jtm) [35]	73.4
RGB, Optical Flow (OF), and Pose-Baseline [20]	76.9
RGB, OF, and Pose-Chained [20]	80.8
DDI, DDNI, and DDMNI [9]	87.8
Fused View-Invariant and Difference Handcrafted cues (FVDH) [36]	81.3
MF-NET [37]	90.0
Multi-stream enhanced spatiotemporal graph convolutional network (MS-ESTGCN) [38]	91.4
Action Machine [39]	94.3
Our EGTSN	94.7
